# Impact of a multidomain lifestyle intervention on regional spontaneous brain activity

**DOI:** 10.3389/fnagi.2022.926077

**Published:** 2022-07-28

**Authors:** So Young Moon, Seong A. Shin, Jee Hyang Jeong, Chang Hyung Hong, Yoo Kyoung Park, Hae Ri Na, Hong-Sun Song, Hee Kyung Park, Muncheong Choi, Sun Min Lee, Buong-O Chun, Jong-Min Lee, Seong Hye Choi

**Affiliations:** ^1^Department of Neurology, Ajou University School of Medicine, Suwon-si, South Korea; ^2^Department of Biomedical Engineering, Hanyang University, Seoul, South Korea; ^3^Department of Neurology, Ewha Womans University School of Medicine, Seoul, South Korea; ^4^Department of Psychiatry, Ajou University School of Medicine, Suwon-si, South Korea; ^5^Department of Medical Nutrition, Graduate School of East-West Medical Nutrition, Kyung Hee University, Suwon-si, South Korea; ^6^Department of Neurology, Bobath Memorial Hospital, Seongnam-si, South Korea; ^7^Department of Sports Sciences, Korea Institute of Sports Science, Seoul, South Korea; ^8^Department of Sports & Health science, Shinhan University, Uijeongbu-si, South Korea; ^9^Graduate School of Physical Education, College of Arts and Physical Education, Myongi University, Yongin-si, South Korea; ^10^Department of Neurology, Inha University School of Medicine, Incheon, South Korea

**Keywords:** regional homogeneity, dementia, prevention, lifestyle, intervention, cognition, SUPERBRAIN, brain activity

## Abstract

In the SoUth Korean study to PrEvent cognitive impaiRment and protect BRAIN health through lifestyle intervention in at-risk elderly people (SUPERBRAIN), we evaluated the impact of multidomain lifestyle intervention on regional homogeneity (ReHo) in resting-state functional brain magnetic resonance imaging (MRI) data. Of 152 participants aged 60–79 years without dementia assigned to either facility-based multidomain intervention (FMI), home-based MI, or controls, we analyzed 56 scanned MRIs at baseline and 24 weeks. ReHo values from regions with significant longitudinal changes were compared between the intervention and control groups and their correlations with the Repeatable Battery for the Assessment of Neuropsychological Status (RBANS) or serum brain-derived neurotrophic factor (BDNF) were evaluated. ReHo values in the left medial orbitofrontal gyrus and right superior parietal lobule were increased [*p* = 0.021, correlated positively with serum BDNF changes (*r* = 0.504, *p* = 0.047)] and decreased [*p* = 0.021, correlated negatively with changes in the total (*r* = −0.509, *p* = 0.044) and attention (*r* = −0.562, *p* = 0.023). RBANS], respectively, in the participants assigned to the FMI group than those of the controls. Our results suggest that facility-based group preventive strategies may have cognitive benefits through neuroplastic changes in functional processing circuits in the brain areas which play a crucial role in the adaptive learning and internally directed cognition.

## Introduction

Recent studies report that multidomain lifestyle interventions including dietary counseling, physical exercise, cognitive training, and vascular/metabolic risk monitoring can yield cognitive benefit in at-risk people. The Finnish Geriatric Intervention Study to Prevent Cognitive Impairment and Disability (FINGER) (Ngandu et al., 2015) exploratory subgroup analyses in the French Multidomain Alzheimer Preventive Trial (MAPT) (Andrieu et al., 2017; Chhetri et al., 2018), the Dutch Prevention of Dementia by Intensive Vascular Care (PreDIVA) (Moll van Charante et al., 2016) studies, or the SoUth Korean study to PrEvent cognitive impaiRment and protect BRAIN health through lifestyle intervention in at-risk older adults (SUPERBRAIN) (Moon et al., 2021) proved the cognitive benefits of a multidomain lifestyle intervention in participants with increased risk of dementia. Such interventions may increase cognitive reserve and reduce inflammation and vascular/oxidative damage in the brain (Livingston et al., 2017, 2020). Cognitive reserve is responsible for the efficiency of the brain functions and its neural substrate is referred to brain volume, cerebral metabolism, or the neural networks such as density of synapses and dendrites branching. Therefore, enhanced cognitive reserve after multidomain lifestyle interventions could be investigated by changes in structural or functional brain imagings or concentration of neurotrophic factors such as the brain-derived neurotrophic factor (BDNF). The exploratory analysis of the SUPERBRAIN (Moon et al., 2021) previously showed that serum BDNF levels were significantly increased in the facility-based multidomain intervention (FMI) compared to the control group. However, benefits on structural or functional brain imagings of multidomain lifestyle interventions remain unclear. The FINGER MRI exploratory sub-study did not reveal significant differences between the intervention and control groups in changes in regional brain volumes, cortical thickness, and white matter lesion volume after 2 years in at-risk elderly without substantial impairment (Stephen et al., 2019). In another FINGER MRI exploratory sub-study using diffusion tensor imaging (DTI), fractional anisotropy decreased to a greater extent in the intervention group than in the control group, with no significant intergroup differences for changes in diffusivity along domain and non-domain diffusion orientations, mean diffusivity, axial diffusivity, or radial diffusivity (Stephen et al., 2020). Furthermore, to our best knowledge, there are no studies on the impact of multidomain lifestyle interventions on the regional networks of resting-state functional magnetic resonance imaging (rs-fMRI) data in at-risk older adults without substantial impairment.

The rs-fMRI is a non-invasive neuroimaging technique for investigating spontaneous brain activity *in vivo* (Smith, 2012). The regional homogeneity (ReHo) and the amplitude of low-frequency fluctuations (ALFFs) are common approaches for depicting the regional characteristics of rs-fMRI data. The ReHo calculates the synchronization of low-frequency fluctuations between a given voxel and the neighboring voxels (Zang et al., 2004), reflecting the neural function synchronization in the local brain region. ALFF measures the ALFFs of individual voxels (Zang et al., 2007), characterizing spontaneous neural activity in the local brain region. Early pathological changes in regional spontaneous brain activity among the Alzheimer's disease (AD) spectrum have been studied using the ReHo and ALFFs (Dai et al., 2012; Long et al., 2016; Ni et al., 2016). Therefore, it would be possible to study the impact of multi-domain lifestyle interventions on the cognitive reserve through the change of the ReHo and ALFFs at risk of dementia.

In this study, we evaluated the impact of a 6-month multidomain lifestyle intervention on changes in ReHo and ALFFs of rs-fMRI using data from the SUPERBRAIN.

## Methods

### Study population

**Figure 1:**
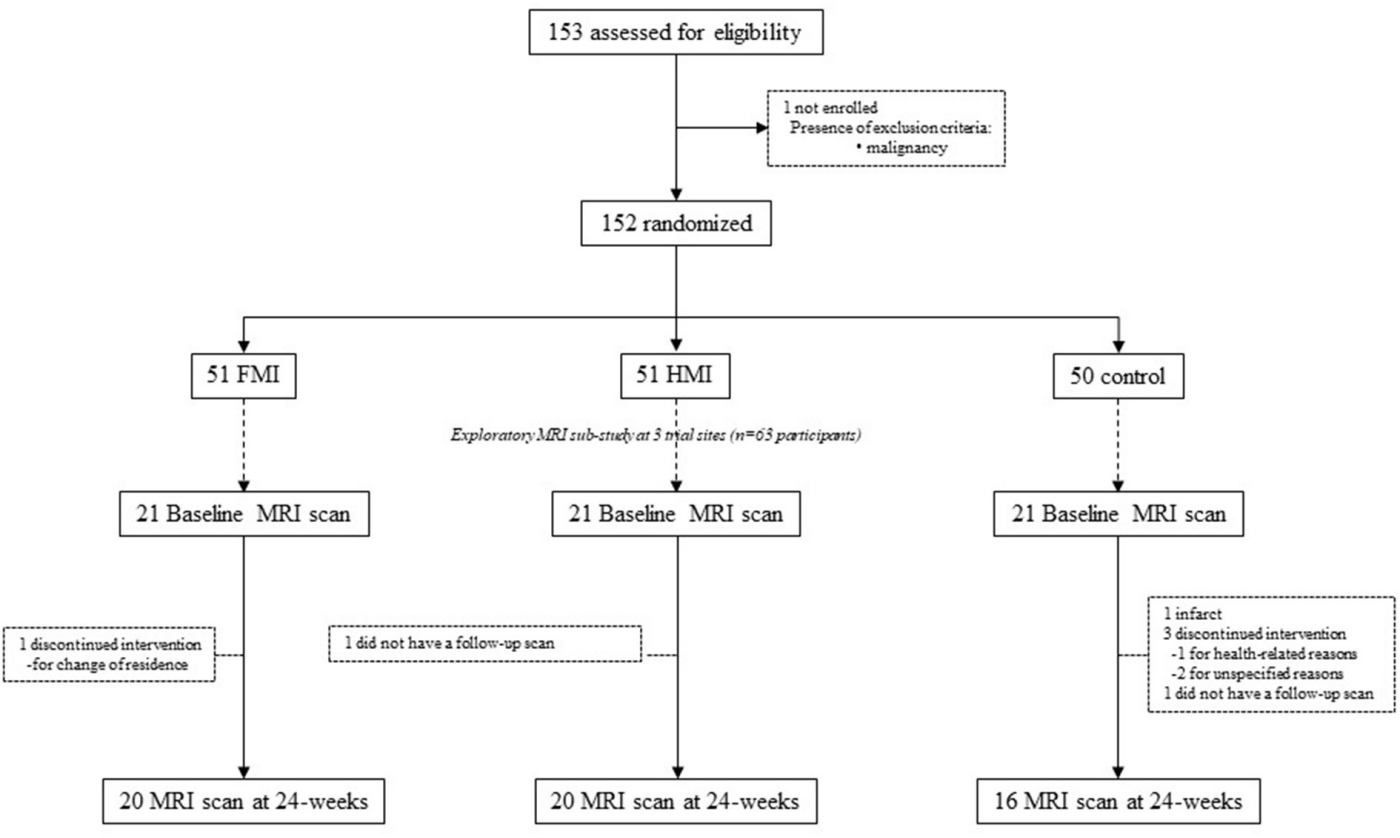
Diagram depicting the exploratory MRI analyses in the SUPERBRAIN trial. SUPERBRAIN, South Korean Study to Prevent Cognitive Impairment and Protect Brain Health Through Lifestyle Intervention; MRI, magnetic resonance imaging; FMI, facility-based multidomain intervention; HMI, home-based multidomain intervention.

The SUPERBRAIN trial protocol (ClinicalTrials.gov: NCT03980392) (Park et al., 2020) and primary findings (Moon et al., 2021) have been described previously. Briefly, this was a 24-week, multicenter, outcome assessor-blinded, randomized controlled trial with a three-parallel-arm design performed at eight medical centers across South Korea. The study included the facility-based multidomain intervention (FMI), home-based multidomain intervention (HMI), and control groups. The participants included 152 individuals aged 60–79 years who had no dementia but had one or more modifiable dementia risk factors such as hypertension, diabetes mellitus, dyslipidemia, smoking, obesity, abdominal obesity, metabolic syndrome, educational level of ≤ 9 years, social isolation, and physical inactivity. In addition, they had a Mini-Mental State Examination z score of ≥-1.5, were able to perform independent activities of daily living and pass a literacy test, and had a reliable informant who can provide investigators with the requested information. Individuals with dementia, conditions affecting safe participation or cooperation such as major psychiatric illness, other neurodegenerative disease, malignancy within the previous 5 years, cardiac stent or revascularization within the previous 1 year, serious or unstable symptomatic cardiovascular disease, other serious or unstable medical disease, severe loss of vision or hearing or communication ability, or concurrent participation in another trial were excluded. This study was conducted in accordance with the International Conference on Harmonization Good Clinical Practice Guidelines. The institutional review boards of all institutions approved the protocol and consent forms before the start of the study. Written informed consent was obtained from all potential participants prior to enrollment. The SUPERBRAIN MRI exploratory sub-study included 63 participants from three trial sites (Ajou University Hospital, Ewha Womans University Medical Center, and Inha University Hospital) (Figure 1). Brain scans were conducted at baseline and 24-week visits. The present study included 56 participants with both baseline and repeat scans of good quality.

### Randomization and intervention

The participants were randomly assigned to the FMI, HMI, and control groups at baseline at a 1:1:1 ratio. The participants from the FMI and HMI groups received interventions of the five components previously described in detail (Park et al., 2020; Moon et al., 2021), including the monitoring and management of metabolic and vascular risk factors, cognitive training and social activity, physical exercise (Lee et al., 2020), nutritional guidance, and motivational enhancement.

The metabolic and vascular risk factors were assessed by blood tests and anthropometric measurements before the intervention. Hypertension, diabetes mellitus, dyslipidemia, obesity, abdominal obesity, smoking, and high alcohol consumption were monitored and managed. Each participant met a study doctor at baseline and week 12. The study doctors informed participants of their risk factors, and prescribed medications if necessary. Participants were educated at baseline by a study nurse and given education booklets describing their risk factors and a booklet of lifestyle guidelines to prevent dementia. They also met the study nurse every 4 weeks for anthropometric measurements and the monitoring of the smoking status and alcohol consumption. Measurements were recorded at each visit in the participants' notebooks for SUPERBRAIN. If the risk factors for a participant did not improve, the study nurse again educated the participant at week 12 with the aid of the booklets. The participants in the FMI group received facility-based interventions three times/week. The HMI group received the same cognitive training and physical exercise programs as the FMI group. The HMI group participated in one group-based cognitive training session (50 min) and one home-based cognitive training session (30–40 min) per week and one group exercise session (60 min) and two home-based exercise sessions (60 min) per week during the first 2 months of the trial. For the remainder of the 6-month study, the HMI group attended one group cognitive training session and one group exercise session every 2 weeks. For the weeks that included group sessions, the participants attended one cognitive training session and two exercise sessions individually at home each week. For weeks that did not include group sessions, participants attended two cognitive training sessions and three exercise sessions alone at home each week. Cognitive training targeted the cognitive domains of episodic memory, executive function, attention, working memory, calculation, and visuospatial function. Cognitive training in group sessions were conducted by trained health professionals (psychologists, occupational therapists, and study nurses) using a tablet-based application. If a participant had difficulty in using a tablet, they were provided with workbooks to be completed under the supervision of trained health professionals. The content of the workbook is similar to the cognitive training application. The physical exercise program consisted of aerobic exercise, exercise to enhance balance and flexibility, muscle-strengthening activities involving major muscle groups, and finger-and-toe movements. The exercise programs in group session were guided by trained exercise professionals at a gym. The physical exercise programs used portable tools such as elastic bands, nine floor plates with numbers, and chairs. Every 2 months, the exercise intensity increased. During home-based exercise sessions, participants exercised at home watching videos on the tablet PC. If participants had difficulty using the tablet, they exercised at home while following instructions on a poster or in a booklet. Participants were given appropriate equipment to enable them to perform the exercises. The nutritional intervention included three individual counseling sessions (each lasting 30 min) and seven group sessions (each lasting 50 min) led by study nutritionists. Individual sessions included tailoring the participant's daily diet and providing education on customized diets to manage individual vascular risk factors. Group sessions provided discussions, practical exercises for facilitating changes in diet, and advice on how to make meals with recommended ingredients *via* a cooking lesson. Motivational enhancement included four group counseling sessions lasting 50 min each and led by a psychologist (at 1, 2, 13, and 24 weeks). Group meetings provided information and support for facilitating dementia prevention program activities, and include discussions on the importance of change, ambivalence, and self-efficacy, as well as family education using video clips. In addition, through the family-coach program, a family member participated in reinforcing the motivation of a participant. Encouraging pop-up video messages made by participants' families and self-rated achievement pop-up messages were provided every week before the tablet-based cognitive intervention. Participants in workbook-type cognitive interventions were sent the encouraging pop-up video messages on their cell phones and performed self-rated achievement assessments on paper. The control group received regular health advice according to the established guidelines.

### Cognitive outcomes and serum brain-derived neurotrophic factor (BDNF)

The total scale index score of the Repeatable Battery for the Assessment of Neuropsychological Status (RBANS) was assessed at baseline and within 4 weeks post-intervention. Before initiating the study, all assessors participated in a workshop to receive education on how to perform outcome measurements (Park et al., 2020). The RBANS consists of tests A, B, C, and D of identical degree of difficulty. Each test includes 12 subtests and evaluates the following five cognitive domains: attention, language, visuospatial/constructional abilities, immediate memory, and delayed memory (Randolph et al., 1998). The participants performed tests A and D of the RBANS at baseline and post-intervention, respectively. A standard score based on same-aged peers for each of the five cognitive domains was obtained. These indices were combined to compute the total scale scores of cognitive functioning. Higher scores indicated better cognitive functioning.

Changes in serum BDNF levels after the multidomain intervention were investigated. Fasting blood samples were collected at ~9 am in serum separator tubes (SSTs) within 4 weeks before the intervention and within 4 weeks after the last intervention. The SSTs were maintained at room temperature for 30 min, centrifuged for 10 min at 3,000 rpm, and sent to a central laboratory. Serum samples were stored at ≤ -70°C until analysis. Serum BDNF levels were measured using a quantitative sandwich enzyme-linked immunosorbent assay (ELISA) (DBD00; R&D Systems, Inc., Minneapolis, MN, USA), according to the manufacturer's instructions.

### MRI acquisition and processing

Brain MRI scans at each clinical site were taken with the following MR systems and rs-fMRI parameters: 3T Achieva, Philips (slice thickness, 4.5 mm; number of slices 31; repetition time (TR), 2,000 ms; echo time (TE), 30 ms; flip angle, 80°; voxel size 3.0 × 3.0 mm^2^ and 176 volumes) at the Ajou University Hospital, 3T Achieva, Philips (slice thickness, 4.5 mm; number of slices 31; TR, 2,000 ms; TE, 30 ms; flip angle, 80°; voxel size 3.0 × 3.0 mm^2^ and 176 volumes) at the Ewha Woman's University Medical Center, and 3T Signa Architect, GE, at the Inha University Hospital (slice thickness, 4.5 mm; number of slices 31; TR, 2,000 ms; TE, 30 ms; flip angle, 90°; voxel size 1.875.0 × 1.875 mm^2^ and 180 volumes). The same imaging parameters and MRI scanners were used for the baseline and 24-week scans. Regular phantom scans were performed within each site, although the phantom was not shared among three centers. Functional data were preprocessed using Data Processing Assistant for rs-fMRI Advanced Edition (DPARSFA) v5.1 and Statistical Parametric Mapping (SPM) 12 implemented on Matlab 2020b (Mathworks, http://www.mathworks.com). The first five volumes were discarded to ensure magnetization equilibrium, and the functional data were corrected for slice timing using the middle volume as a reference. Then, the data were spatially realigned to the middle slice using a least-squares approach and six-parameter rigid body spatial transformations, and no subjects showed excessive head motions >2.5 mm and 2.5° in any dimensions during the scan acquisition. The realigned data were spatially normalized to a Montreal Neurological Institute (MNI) standard space using an echo planar image template and smoothed with a Gaussian kernel of 4-mm full-width-half-maximum, followed by the removal of linear trend and regression of covariates including Friston 24-motion parameters and signals from the white matter and cerebrospinal fluid. Calculations for ALFF and ReHo were performed using DPARSFA software. The ALFF value was calculated by obtaining a power spectrum *via* a Fast Fourier Transform and averaging the square root of the power spectrum at the frequency bands of 0.01–0.08 Hz at each voxel. Then, the ALFF value of each voxel was modulated by dividing it by global mean ALFF. Prior to ReHo calculation, temporal band-pass filtering at 0.01–0.1 Hz was applied to spatially normalized functional data. ReHo was calculated by applying Kendall's Coefficient of Concordance which calculates the similarity of the time series of a voxel with that of 27 neighboring voxels. The ReHo at each voxel was divided by the global mean ReHo, and the individual modulated ReHo maps were spatially smoothed with a Gaussian kernel of 4-mm full-width-half-maximum.

### Statistical analysis

Group differences at baseline were analyzed by *t*-test (MRI vs. non-MRI groups) or one-way analyses of variance (ANOVA, FMI vs. HMI vs. controls) for independent samples and the chi-square test for categorical variables. In addition, two-way ANOVA, with time and groups as independent factors, was used to compare changes in the RBANS and serum BDNF levels from baseline to the study endpoint. The groups were considered similar when *p* > 0.05. *Post-hoc* analyses were done by Dunnett test.

Individual ALFF and ReHo maps at baseline were compared among groups using one-way analyses of covariance (ANCOVA) with age, sex, years of education, and imaging site effects added as covariates of no interest. In addition, to compare changes in the ALFF and ReHo maps from baseline to the study endpoint, we used two statistical analyses. First, two-way ANCOVA was used, with time and groups as independent factors and age, sex, years of education, and imaging site effects added as covariates of no interest. Secondly, voxel-wise paired *t*-tests were employed to analyze longitudinal changes in spontaneous brain activity for each group, using age, sex, years of education, and site effects as covariates. The statistical significance level was set at family-wise error corrected *p*-value <0.05 at the cluster level. ALFF and ReHo values were compared among groups using two-sample *t*-tests (FMI vs. controls and HMI vs. controls). Finally, correlations between clinical parameters (total and standard scores cognitive domains of the RBANS) or the serum BDNF level and changes in ALFF or ReHo were evaluated at a statistical significance level of p <0.05 (uncorrected for multiple corrections).

## Results

The population undergoing MRI was younger, more educated, and had a higher baseline RBANS total scale index than the population not undergoing MRI in SUPERBRAIN. However, there were no differences in intervention adherence, sex distribution, APOE ε4 carriers, and group distribution between the two populations (Supplementary Table 1). The intervention and control groups in the SUPERBRAIN exploratory MRI sub-study were not significantly different in demographic, clinical, cognitive, and MRI characteristics at baseline (Table 1). The adherence rates in the FMI and HMI groups were 96.0 and 97.0%, respectively.

**Table 1:** Baseline clinical characteristics in the intervention and control groups.

	**FMI (*****n*** = **20)**	**HMI (*****n*** = **20)**	**Control (*****n*** = **16)**	***p*** **-value**
Age, y	68.1 ± 4.5	68.8 ± 4.7	67.3 ± 4.4	0.663
Education, y	12.0 ± 3.7	11.6 ± 4.0	9.8 ± 4.3	0.352
Female, *n* (%)	14 (70.0)	12 (60.0)	14 (87.5)	0.352
APOE ε4 carriers, *n* (%)	3 (15.0)	2 (10.0)	3 (18.8)	0.782
RBANS indexes				
Total	110.1 ± 13.7	109.6 ± 19.1	105.5 ± 18.8	0.682
Attention	113.3 ± 14.2	112.3 ± 16.5	104.8 ± 18.1	0.286
Immediate recall	104.2 ± 16.0	106.1 ± 15.7	101.1 ± 15.2	0.649
Delayed recall	98.2 ± 11.5	99.7 ± 18.3	99.9 ± 17.4	0.944
Visuospatial/construction	99.1 ± 11.1	101.5 ± 14.2	95.6 ± 13.4	0.413
Language	108.7 ± 15.2	111.8 ± 10.3	111.2 ± 15.1	0.752
BDNF, ng/mL	29.9 ± 11.4	29.9 ± 11.4	40.5 ± 22.1	0.168

FMI, facility-based multidomain intervention; HMI, home-based multidomain intervention, RBANS, Repeatable Battery for the Assessment of Neuropsychological Status; BDNF, brain-derived neurotrophic factor.

Changes in the RBANS total scale index scores, attention and visuospatial/construction were different among three groups (Table 2). *Post-hoc* analyses revealed that the FMI groups showed significantly improved post-intervention RBANS total scale index scores (*p* = 0.047), attention (*p* = 0.016) and visuospatial/construction (*p* = 0.009) compared to the control group. Serum BDNF levels did not significantly change among groups (Table 2).

**Table 2:** Changes in clinical characteristics after 24 weeks in the intervention and control groups.

	**FMI (*****n*** = **20)**	**HMI (*****n*** = **20)**	**Control (*****n*** = **16)**	***p*** **-value**		
				**Group**	**Time**	**Group** *time
RBANS indexes						
Total	7.1 ± 6.4	5.2 ± 9.7	−2.3 ± 9.8	0.047	0.295	0.452
Attention	1.5 ± 8.2	−0.5 ± 9.0	−2.7 ± 9.1	0.011	0.866	0.837
Immediate recall	6.2 ± 9.4	7.3 ± 14.1	3.3 ± 10.4	0.209	0.072	0.902
Delayed recall	10.6 ± 10.3	11.0 ± 9.3	2.9 ± 7.9	0.572	0.004	0.446
Visuospatial/construction	3.0 ± 9.3	−0.9 ± 16.4	−8.4 ± 13.4	0.006	0.366	0.202
Language	2.1 ± 12.1	0.1 ± 11.5	−3.1 ± 11.1	0.811	0.964	0.649
BDNF, ng/mL	14.7 ± 24.6	4.2 ± 2.2	−3.7 ± 2.6	0.744	0.100	0.088

FMI, facility-based multidomain intervention; HMI, home-based multidomain intervention, RBANS, Repeatable Battery for the Assessment of Neuropsychological Status; BDNF, brain-derived neurotrophic factor.

### Longitudinal changes in ALFF

The spontaneous functional activity represented by ALFF was comparable among the groups at baseline. In the first statistical analysis using two-way ANCOVA, any significant difference in longitudinal changes in the ALFF maps was not revealed. In the second statistical analysis which analyze longitudinal changes in spontaneous brain activity for each group, we did not detect any longitudinal changes in ALFF in the HMI and control groups. However, a significant increase in ALFF in the left medial orbitofrontal gyrus was observed at follow-up in the FMI group (Supplementary Table 2 and Supplementary Figure 1). The ALFF in the left medial orbitofrontal gyrus in the FMI group tended to be more increased than that in the control group at follow-up (mean ± standard error, 0.312 ± 0.060 vs. 0.101 ± 0.069, *p* = 0.083). Longitudinal changes in ALFF in the left medial orbitofrontal gyrus were not different between the HMI and control groups (mean ± standard error, −0.013 ± 0.060 vs. 0.101 ± 0.069, *p* = 0.691). Averaged ALFF images in each group at baseline or follow-up were provided in the Supplementary Figure 2.

### Longitudinal changes in ReHo

**Figure 2:**
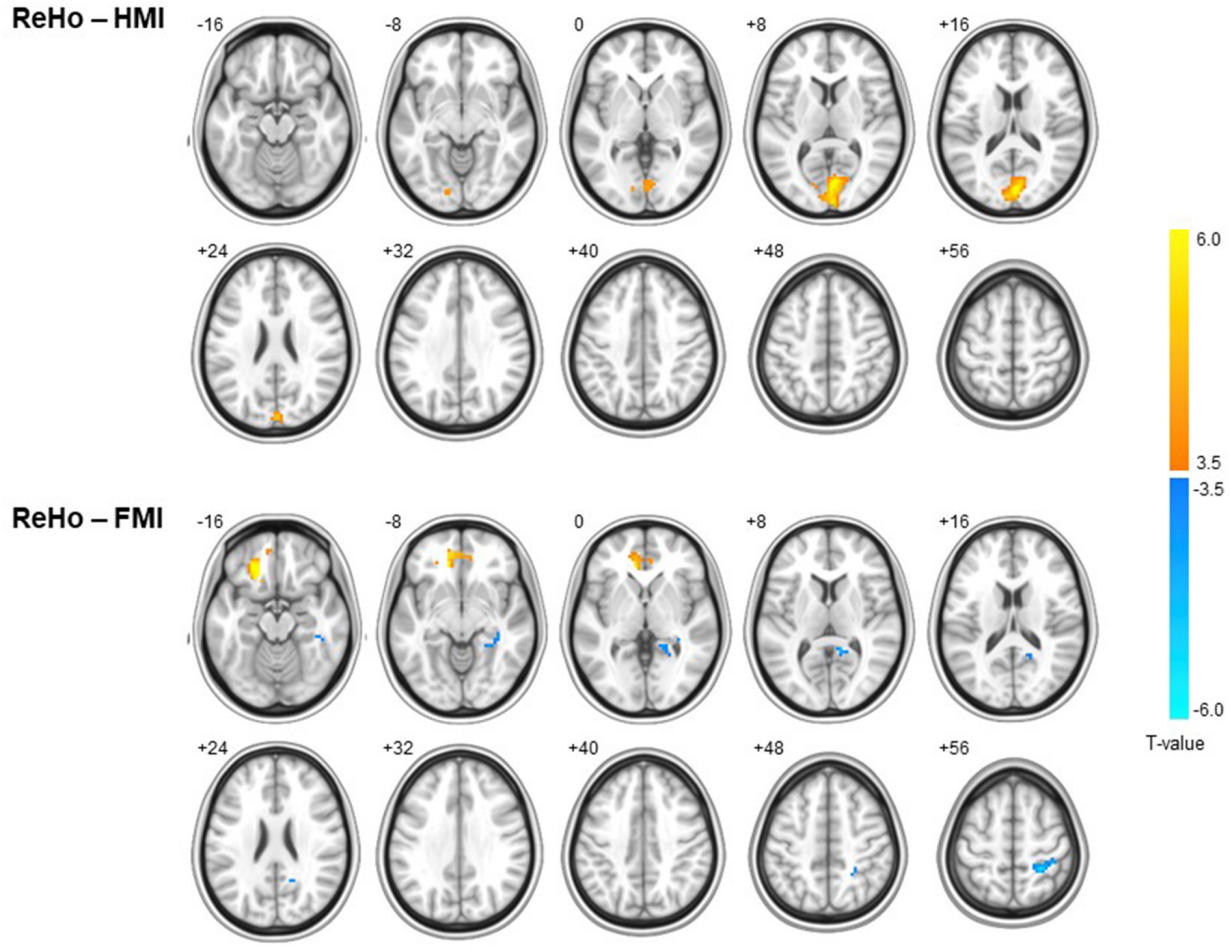
Within-group differences in ReHo in the HMI (upper panel) and FMI groups (lower panel). A warm color indicates increased ReHo at follow-up, and a cool color indicates decreased ReHo. In the HMI group, increased ReHo was evident only in the cuneus at follow-up, whereas increased ReHo in the medial orbitofrontal gyrus and anterior cingulate and decreased ReHo in right superior parietal lobule and precuneus were observed in the FMI group. FMI, facility-based multidomain intervention; HMI, home-based multidomain intervention; ReHo, regional homogeneity.

One-way ANOVA revealed no group differences in ReHo at baseline. In the first statistical analysis using two-way ANCOVA, any significant difference in longitudinal changes in the ReHo maps was not revealed. In the second statistical analysis which analyze longitudinal changes in spontaneous brain activity for each group, in the HMI group, increased ReHo was evident only in the right cuneus at follow-up, whereas increased ReHo in the left medial orbitofrontal gyrus and left anterior cingulate and decreased ReHo in the right superior parietal lobule and right precuneus were observed in the FMI group (Figure 2 and Table 3). However, longitudinal alterations in ReHo were not found in the control group. The ReHo change (follow-up ReHo - baseline ReHo) in the right cuneus was significantly higher in the HMI group than in the control group (mean ± standard error, 0.204 ± 0.046 vs. 0.001 ± 0.052, *p* = 0.017). There was a significant difference between the FMI and control groups in ReHo changes in the left medial orbitofrontal gyrus (mean ± standard error, 0.244 ± 0.056 vs. −0.001 ± 0.065, *p* = 0.021) and the right superior parietal lobule (mean ± standard error, −0.182 ± 0.038 vs. −0.015 ± 0.044, *p* = 0.021). However, the difference in ReHo changes in the left anterior cingulate (mean ± standard error, 0.225 ± 0.052 vs. 0.077 ± 0.060, *p* = 0.217) and the right cuneus (mean ± standard error, −0.150 ± 0.021 vs. −0.078 ± 0.024, *p* = 0.090) was not significant between the FMI and control groups. Averaged ReHo images in each group at baseline or follow-up were provided in the Supplementary Figure 3.

**Table 3:** Brain regions with significant longitudinal changes in ReHo.

**Group**	**Contrast**	**Brain region** ^*^	**Cluster size**	**Peak**, ***T*****-value**	**MNI coordinate**		
					**x**	**y**	**z**
Control	Baseline < Follow-up	–					
	Baseline > Follow-up	–					
HMI	Baseline < Follow-up	Cuneus, R	329	6.84	3	−84	15
				5.90	6	−96	6
				5.31	12	−81	6
	Baseline > Follow-up	–					
FMI	Baseline < Follow-up	Medial orbital gyrus, L	101	7.33	−21	30	−15
				5.73	−24	30	−24
				3.98	−18	48	−12
		Anterior cingulate gyrus, L	106	6.42	−12	45	−6
				5.73	−9	36	0
				4.14	9	45	−9
	Baseline > Follow-up	Superior parietal lobule, R	126	6.73	27	−42	63
				3.97	24	−48	45
				3.90	39	−30	51
		Precuneus, R	84	5.22	18	−54	21
				4.70	36	−33	−3
				4.65	12	−45	9

* Results at family-wise error corrected p-value <0.05 at cluster level.

FMI, facility-based multidomain intervention; HMI, home-based multidomain intervention; L, left; MNI, Montreal Neurological Institute; R, right; ReHo, regional homogeneity.

### Correlations between ALFF and ReHo and clinical variables

**Figure 3:**
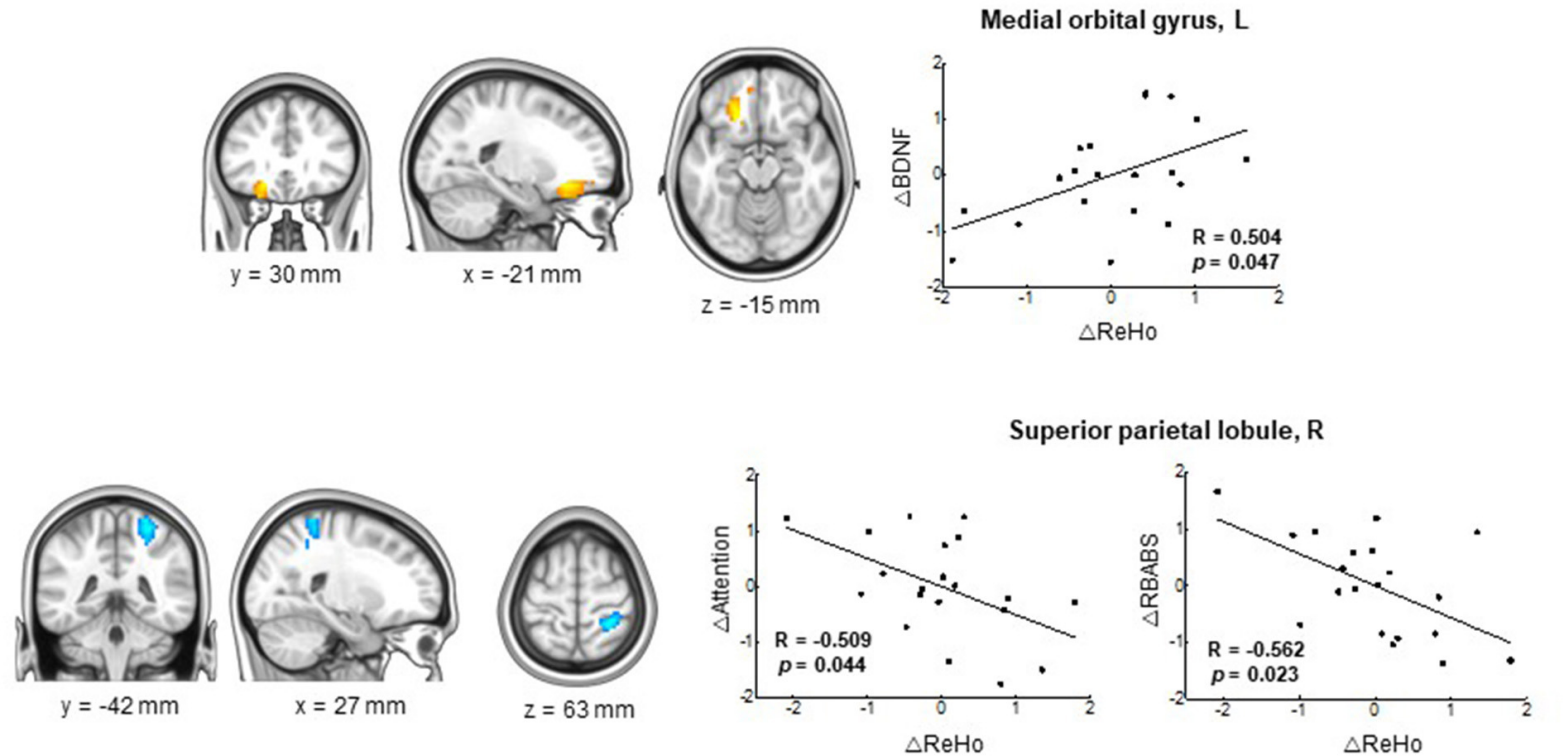
Correlations between longitudinal changes in ReHo and RBANS score and serum BDNF in the FMI group. Significant correlation was found between the serum BDNF and longitudinal changes in ReHo in the left medial orbital gyrus (upper panel) and between the attention/RBANS total scores and longitudinal changes in the ReHo in the right superior parietal lobule (lower panel) in the FMI group. A positive correlation was found between longitudinal changes in ReHo in the left medial orbitofrontal gyrus and BDNF in the FMI group. Negative correlations were found between longitudinal changes in ReHo in the right superior parietal lobule and attention/RBANS total scores in the FMI group. BDNF, brain-derived neurotrophic factor; FMI, facility-based multidomain intervention; HMI, home-based multidomain intervention; RBANS, Repeatable Battery for the Assessment of Neuropsychological Status; ReHo, regional homogeneity.

Correlations between ALFF and ReHo and clinical variables were evaluated in participants with longitudinal changes in spontaneous regional activity. In the FMI group, ALFF changes in the left medial orbitofrontal gyrus did not correlate significantly with clinical data, though longitudinal changes in ReHo in the left medial orbitofrontal gyrus correlated positively with BDNF (Figure 3 and Table 4). Negative correlations were found between the longitudinal changes in ReHo in the right superior parietal lobule and the attention and total RBANS scores in the FMI group, and between the longitudinal changes in ReHo in the right cuneus and delayed memory in the HMI group (*r* = −0.538, *p* = 0.032).

**Table 4:** Correlation between clinical variables and longitudinal changes in ReHo in regions with significant longitudinal changes in the FMI group.

**Brain region**	**Measure**	**BDNF**	**Immediate memory**	**Delayed memory**	**Visuocon-struction**	**Language**	**Attention**	**Total RBANS**
Medial orbital gyrus, L	*r*	0.504^*^	0.084	0.347	−0.258	−0.069	0.260	0.165
	*p*-value	0.047^*^	0.756	0.187	0.336	0.800	0.331	0.542
Anterior cingulate, L	*r*	0.398	0.324	0.451	0.124	−0.136	0.268	0.429
	*p*-value	0.127	0.220	0.080	0.648	0.615	0.316	0.097
Superior parietal lobule, R	*r*	−0.101	−0.276	−0.198	−0.175	−0.093	−0.509^*^	−0.562^*^
	*p*-value	0.710	0.302	0.463	0.518	0.733	0.044^*^	0.023^*^
Precuneus, R	*r*	−0.308	−0.333	−0.089	0.097	−0.277	0.020	−0.317
	*p*-value	0.247	0.208	0.744	0.720	0.298	0.943	0.232

* Significant at p-value < 0.05.

BDNF, brain-derived neurotrophic factor; FMI, facility-based multidomain intervention; HMI, home-based multidomain intervention; L, left; R, right; RBANS, Repeatable Battery for the Assessment of Neuropsychological Status; ReHo, regional homogeneity.

## Discussion

To our best knowledge, this is the first study to reveal that a multidomain lifestyle intervention for dementia prevention could be beneficial on regional spontaneous brain activity. In the FMI group, the post-intervention increase in ReHo in the left medial orbitofrontal gyrus was significantly higher than that in the control group and correlated positively with changes in serum BDNF. Additionally, in the FMI group, the post-intervention decrease in ReHo in the right superior parietal lobule was significantly higher than that in the control group and correlated negatively with changes in the attention and total RBANS scores. Finally, in the HMI group, the post-intervention ReHo in the right cuneus was significantly increased compared to that in the control group and correlated negatively with changes in the delayed memory score. Therefore, in this study, enhanced cognitive reserve after the facility-based multidomain lifestyle intervention could be revealed by changes in functional brain imaging for regional spontaneous brain activity or concentration of the BDNF.

ReHo, which is based on the similarity of neighboring voxels over a time series, can be used to determine localized functional changes in the brain. It is highly reliable for studying the consistency of local brain activity (Zang et al., 2004; Li et al., 2020). Local brain activity may be altered in various ways across the Alzheimer's disease (AD) spectrum. A recent activation likelihood estimation meta-analysis including 30 functional neuroimaging studies in a salience network in mild cognitive impairment (MCI) revealed a decreased ReHo in the anterior cingulate gyrus in amnestic MCI (Song et al., 2021). A recent study reported that patients with AD showed a significant decrease in ReHo in the right posterior cingulate cortex (a hub region of the default mode network) compared to healthy controls (Zhang et al., 2021). These alternations in local brain activity may be novel targets for formulating interventions to delay AD progression of health. In agreement with previous studies, our study showed that a multidomain lifestyle intervention could alter local brain connectivity, and that these changes correlated in a beneficial way with changes in the serum BDNF and cognitive outcomes in the FMI group.

In prodromal AD patients, ReHo is reported to increase or decrease according to the resilience of the anti-amyloidosis mechanisms. One study revealed that ReHo was significantly higher in the bilateral precuneus (a hub region of the default mode network) for amyloid-positive subjective cognitive decline (SCD) compared to amyloid-negative SCD (Li et al., 2021), whereas other studies reported that ReHo decreases in the left precuneus in prodromal AD (Kang et al., 2017). Therefore, alterations in ReHo may be interpreted in the context of other outcomes. In our study, there was a post-intervention increase in ReHo in the left medial orbitofrontal gyrus in the FMI group, which correlated positively with serum BDNF level changes. Interestingly, one human study showed that *BDNF* polymorphism influenced working memory performance and ReHo in working memory-related brain areas including the left medial frontal gyrus and right medial orbitofrontal gyrus (Chen et al., 2016); another study revealed that physical exercise in mice increased ReHo in the limbic system and improved hippocampal BDNF levels (Dong et al., 2020). Although the increased ReHo in the left orbitofrontal gyrus in the FMI group did not correlate with cognitive outcomes in our study, it correlated with the changes in BDNF levels. Therefore, considering previous studies, increased ReHo in the left orbitofrontal gyrus in the FMI group could be interpreted to be beneficial and may prevent cognitive decline. In the FMI group, the post-intervention decrease in ReHo in the right superior parietal lobule correlated negatively with changes in the attention and RBANS total scores. This may signify a benefit with reference to cognitive improvement. However, in the HMI group, the post-intervention increase in ReHo in the right cuneus correlated negatively with changes in the delayed memory score, which was not beneficial. Therefore, in our study, changes in the regional brain connectivity after the multidomain life intervention occurred in beneficial way in the FMI group, in contrast to that in the HMI group.

In the FMI group, the ReHo in the left orbitofrontal gyrus increased post-intervention. This area is important in signaling the expected rewards/punishments of an action in a given situation (Chen et al., 2016). Accordingly, the brain is capable of comparing the expected reward/punishment with the actual delivery of reward/punishment, thus making the orbitofrontal gyrus critical for adaptive learning. The orbitofrontal gyrus is affected extensively by AD pathology (Van Hoesen et al., 2000). The posterior and medial orbitofrontal areas are the most severely damaged, which suggests that this pathology may contribute heavily to the non-memory-related behavioral changes in AD. However, orbitofrontal areas exhibit a distinct gene expression profile characterized by the relative upregulation of genes implicated in ionotropic and metabotropic neurotransmission as well as the activation of immune responses; the above confers a higher capacity for plastic changes in response to lifetime intellectual enrichment and a potential higher resilience to age-related pathologic brain changes (Bartrés-Faz et al., 2019). In agreement with previous studies, the FMI applied in our study induced increased local connectivity in the medial orbitofrontal areas and may thus be able to prevent non-memory-related behavioral changes and improve adaptive learning. The ReHo in the right superior parietal lobule decreased in the FMI group after the intervention; this area continues onto the medial surface of the hemisphere as the precuneus and is involved in mental imagery and recall of personal experiences. Like the medial prefrontal cortex, it is part of the default mode network of the brain and is engaged during activities such as daydreaming and introspection (Yuan et al., 2021). The decreased ReHo in one component of the default network observed in our study may be interpreted as a shift in brain activation (in an efficient way) from introspection into task-driven activation because the decreased ReHo in this area correlated with cognitive improvement.

In contrast to the FMI, the HMI did not exert beneficial changes in ReHo. In the HMI group, the post-intervention increase in ReHo in the right cuneus correlated negatively with changes in delayed memory. The HMI group participants may spend more time watching the intervention-related tablet/poster than the controls. In our study, the population undergoing MRI was younger, more educated, and had a higher baseline RBANS total scale index than the population not undergoing MRI. In younger and/or intelligent populations, a more powerful intervention might be necessary to provoke plastic changes in the brain. The FMI program involved face-to-face interventions and group-based interactions. Therefore, this FMI could provide more stimuli for plastic changes in the brain than HMI. In future studies on HMI, time spent on passive watching of interventional materials should be reduced, and time spent with other participants/supervisors for social interaction with the objective monitoring of intervention intensity should be increased.

The main strengths of this study are the randomized controlled design, the multidomain intervention, and the availability of MRI scans at baseline and 6 month. The main limitation of this study is that the MRI scanners differed between sites; however, this was adjusted for during analysis. Additionally, as this was a SUPERBRAIN MRI exploratory sub-study, the results should be interpreted with caution. Future studies including regional spontaneous brain activity as a primary outcome measure are necessary to confirm the impact of multidomain lifestyle interventions on regional spontaneous brain activity.

In conclusion, significant changes in regional spontaneous brain connectivity in at-risk elderly without substantial impairment after the FMI suggest that facility-based group preventive strategies may confer cognitive benefits through neuroplastic changes of functional processing circuits in the brain areas which play a crucial role in adaptive learning and internally directed cognition.

## Data availability statement

The original contributions presented in the study are included in the article/Supplementary Material, further inquiries can be directed to the corresponding author/s.

## Ethics statement

The studies involving human participants were reviewed and approved by Inha University Hospital, Ajou University Hospital, and Ewha Womans University Medical Center. The patients/participants provided their written informed consent to participate in this study.

## Author contributions

SM, JJ, CH, YP, J-ML, and SC: conceptualization, methodology, writing-review and editing, and funding acquisition. SM and SS: formal analysis, investigation, and writing-original draft preparation. HN, HP, SL, H-SS, MC, and B-OC: supervision. All authors contributed to manuscript revision, read, and approved the submitted version.

## Funding

This work was supported by grants from the Korea Health Technology R&D Project through the Korea Health Industry Development Institute (KHIDI) and Korea Dementia Research Center (KDRC), funded by the Ministry of Health & Welfare and Ministry of Science and ICT, Republic of Korea (HU20C0198, and HU21C0016). The funders had no role in the study design, data collection, analysis, interpretation of data, writing of the report, or decision to submit the manuscript for publication.

## Conflict of interest

SM received a research grant from Hyundai Pharmaceutical Co. Ltd. CH receives research support from Eisai Korea Inc. JJ receives research grants from Chong Kun Dang Pharmaceutical Corp., Jeil Pharmaceutical Co. Ltd., and Kuhnil Pharmaceutical Co. Ltd., and consults for PeopleBio Co. Ltd. SM, CH, JJ, YP, HN, and SC are shareholders of Rowan Inc. YP consults for Pulmuone Co. Ltd. HN consults for Hyundai Pharmaceutical Co. Ltd. SC consults for Hyundai Pharmaceutical Co. Ltd. and PeopleBio Co. Ltd. The remaining authors declare that the research was conducted in the absence of any commercial or financial relationships that could be construed as a potential conflict of interest.

## Publisher's note

All claims expressed in this article are solely those of the authors and do not necessarily represent those of their affiliated organizations, or those of the publisher, the editors and the reviewers. Any product that may be evaluated in this article, or claim that may be made by its manufacturer, is not guaranteed or endorsed by the publisher.
