# Supplementary material for: Impact of a multidomain lifestyle intervention on regional spontaneous brain activity
Source: Front Aging Neurosci. 2022 Jul 28;14:926077. doi: 10.3389/fnagi.2022.926077 (PMC9366741; doi:10.3389/fnagi.2022.926077)
Supplement: Supplementary file 1 [file Data_Sheet_1.docx]

Supplementary Material

# Supplementary Tables

Table 1. Clinical characteristics participants with or without MRIs

| Parameters | Total | MRI  (n=56) | Non-MRI  (n=96) | P |
| --- | --- | --- | --- | --- |
| Age, y | 152 | 68.2±4.5 | 73.1±3.9 | <0.001 |
| Education, y | 152 | 11.1±4.0 | 9.3±5.1 | 0.015 |
| Female, n (%) | 152 | 38 (69.1) | 74 (76.3) | 0.344 |
| APOE ε4 carriers, n (%) | 151 | 8 (14.8) | 18 (18.6) | 0.656 |
| RBANS at baseline | 152 | 108.6±17.3 | 95.8±17.9 | <0.001 |
| Group (control/FMI/HMI), n (%) | 152 | 16 (29.1)/20 (36.4)/19 (34.5) | 34 (35.1)/31 (32.0)/32 (33.0) | 0.738 |
| Adherence, % | 102 | 96.5±4.6 | 95.1±10.1 | 0.426 |

FMI, facility-based multidomain intervention; HMI, home-based multidomain intervention, RBANS, Repeatable Battery for the Assessment of Neuropsychological Status

Table 2. Brain regions with significant longitudinal changes in ALFF

| Group | Contrast | Brain Region | Cluster size | Peak T-value | MNI coordinate | | |
| --- | --- | --- | --- | --- | --- | --- | --- |
|  |  |  |  |  | x | y | Z |
| Control | Baseline < Follow-up | - |  |  |  |  |  |
|  | Baseline > Follow-up | - |  |  |  |  |  |
| HMI | Baseline < Follow-up | - |  |  |  |  |  |
|  | Baseline > Follow-up | - |  |  |  |  |  |
| FMI | Baseline < Follow-up | Medial orbital gyrus, L | 102 | 4.87 | -18 | 48 | -21 |
|  |  |  |  | 4.75 | -9 | 51 | -18 |
|  |  |  |  | 4.65 | -3 | 45 | -6 |
|  | Baseline > Follow-up | - |  |  |  |  |  |

# Supplementary Figures

**
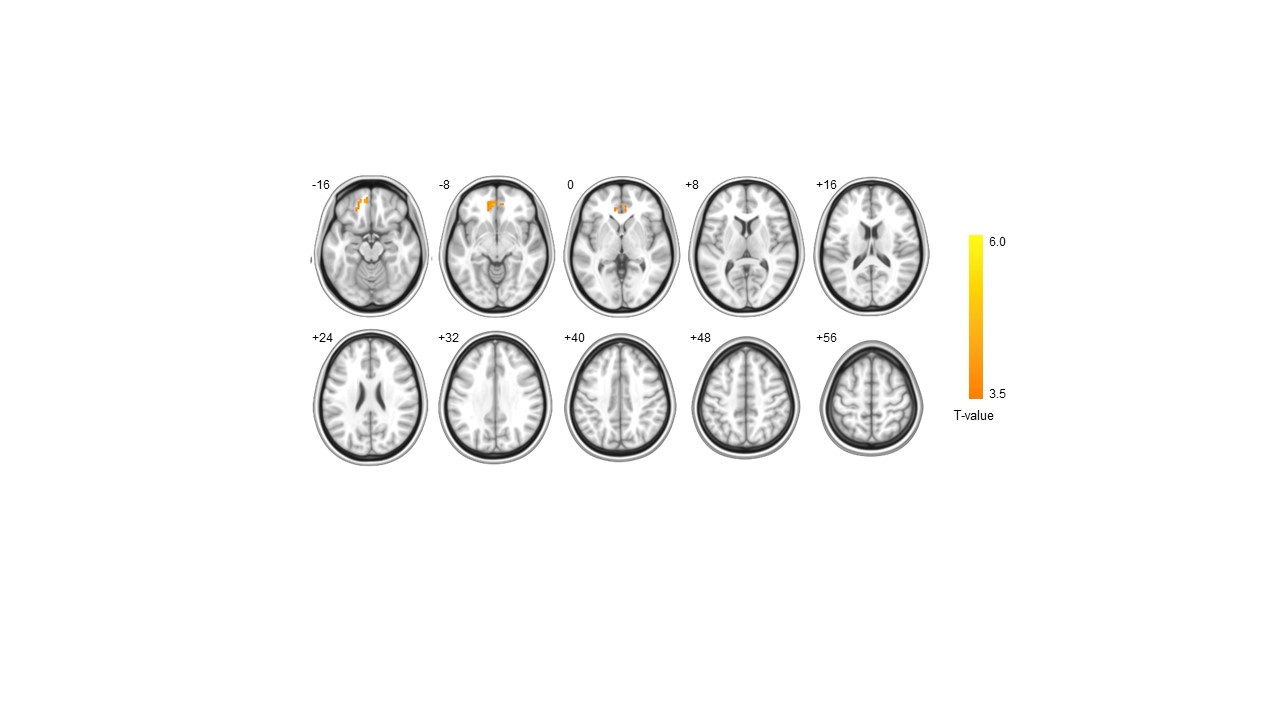
**

**Supplementary Figure 1.** Within-group differences in ALFF in the FMI group. A warm color indicates increased ALFF at follow-up. In the FMI group, a significant increase in ALFF in the medial orbitofrontal gyrus was observed at follow-up. When ALFF differences (follow-up ALFF - baseline ALFF) in the medial orbitofrontal gyrus were compared between the FMI and control groups, the regional ALFF at follow-up in the FMI group was considerably elevated in comparison to the control group but the difference was not significant (mean±standard deviation, 0.312±0.060 vs. 0.101±0.069, *p*=0.083). In addition, the longitudinal changes in ALFF in the medial orbitofrontal gyrus were not different between the HMI and control groups (mean±standard deviation, -0.013±0.060 vs. 0.101±0.069, *p*=0.691).

ALFF, amplitude of low frequency fluctuation; FMI, facility-based multidomain intervention; HMI, home-based multidomain intervention.


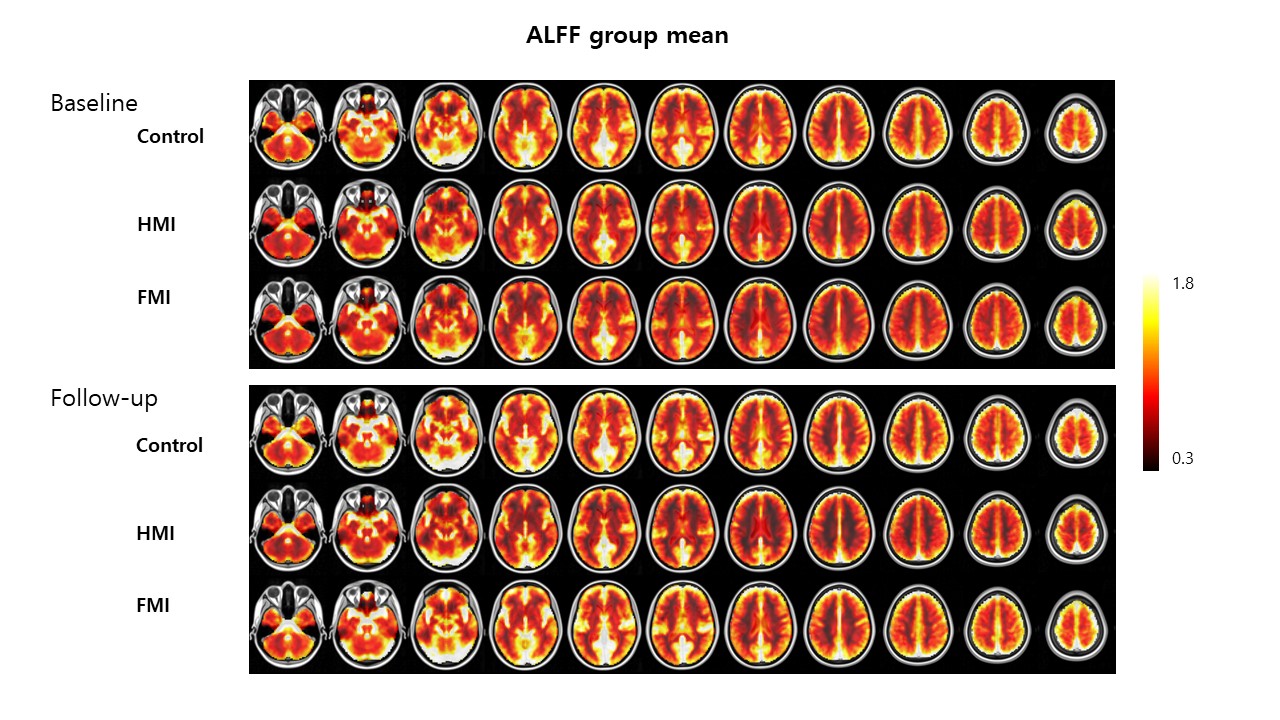


**Supplementary Figure 2.** Averaged ALFF images in each group at baseline or follow-up.

ALFF, amplitude of low frequency fluctuation; FMI, facility-based multidomain intervention; HMI, home-based multidomain intervention.

**Supplementary Figure 3.** Averaged ReHo images in each group at baseline or follow-up.


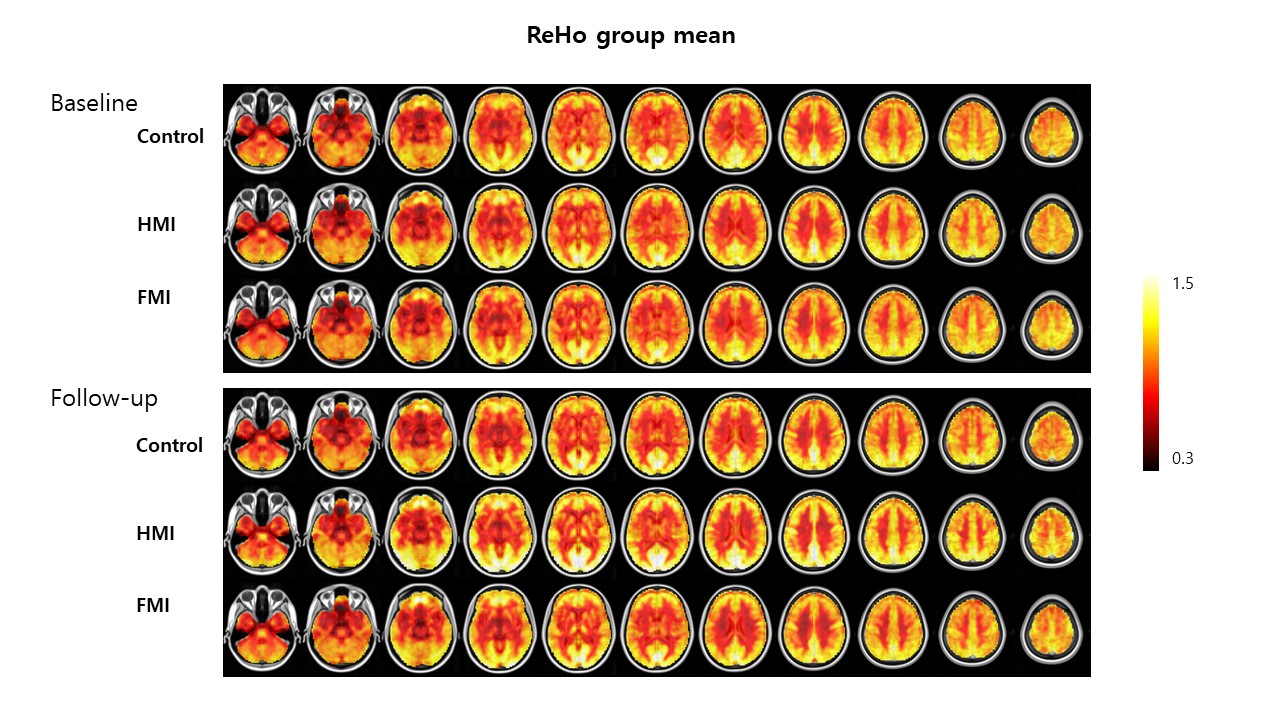


FMI, facility-based multidomain intervention; HMI, home-based multidomain intervention; ReHo, regional homogeneity.
